# Catastrophic Health Expenditure and Its Determinants Among Households With Breast Cancer Patients in China: A Multicentre, Cross-Sectional Survey

**DOI:** 10.3389/fpubh.2021.704700

**Published:** 2021-07-05

**Authors:** Cheng-yao Sun, Ju-fang Shi, Wen-qi Fu, Xin Zhang, Guo-xiang Liu, Wan-qing Chen, Jie He

**Affiliations:** ^1^Department of Health Economics, College of Health Management of Harbin Medical University, Harbin, China; ^2^Office of Cancer Screening, National Cancer Center/National Clinical Research Center for Cancer/Cancer Hospital, Chinese Academy of Medical Sciences and Peking Union Medical College, Beijing, China; ^3^National Cancer Center/National Clinical Research Center for Cancer/Cancer Hospital, Chinese Academy of Medical Sciences and Peking Union Medical College, Beijing, China

**Keywords:** catastrophic health expenditures, insurance, breast cancer, China, economic burden

## Abstract

**Background:** Although numerous studies have examined catastrophic health expenditures (CHE) worldwide, most focus on the general population, not on specific vulnerable groups. We aimed to analyse the extent and the influencing factors of CHE in households with breast cancer patients in China, and explore the ability of different insurances to protect these households from CHE.

**Methods:** A multicentre, cross-sectional interview surveys was conducted in households with breast cancer patients across seven provinces/municipalities in China. CHE were defined as out-of-pocket expenditures ≥ 40% of households' non-food expenditures. Chi-square tests and logistic regression analysis were performed to identify the determinants of CHE in household with breast cancer patients.

**Results:** In the 639 participating households with breast cancer patients, the mean out-of-pocket (OOP) expenditure accounted for ~55.20% of the mean households' non-food expenditures. The overall incidence of CHE was 87.95 and 66.28% before and after insurance compensation, respectively. The logistic regression model revealed that education, disease course, health insurance, treatment method, and income were significant predictors of CHE.

**Conclusions:** The results indicated that medical insurance protects some households with breast cancer patients from the impact of CHE. However, their reimbursement rates were relatively low. Therefore, breast cancer still had a significant catastrophic effect on the economy of households. Policy efforts should focus on improving insurance compensation rates and relieving the economic burden of critical illnesses such as breast cancer.

## Background

According to GLOBOCAN statistics, in 2018, there were 18.1 million new cancer cases and 9.6 million deaths worldwide; among women (8.6 million new cases and 4.2 million deaths), breast cancer was the most commonly diagnosed (24.2%) cancer and the leading cause (15.0%) of cancer deaths (1). In China, breast cancer was also the most commonly diagnosed (1.778 million) cancer and the fifth most common cause of cancer death (0.858 million) among women (2). The high incidence and mortality rates of breast cancer in Chinese women can result in CHE and impose a substantial financial burden.

Despite the considerable improvement in health outcomes, the financial catastrophe caused by the excessive spending on healthcare services appears to be a threat to the health system (3). Generally, healthcare expenditure is considered as “catastrophic” when the OOP health spending greatly exceeds a certain proportion of households' income or capacity to pay (CTP) (4, 5). CHE may lead to a reduction in expenditures on basic commodities (6), even for patients choosing to abandon health care services, thus suffering from illness deterioration (7, 8).

Studies on CHE have been conducted globally, mostly using microdata to evaluate residents' economic burdens. Xu et al. reported that, annually and worldwide, over 150 million people suffer from financial catastrophe and approximately 100 million become impoverished owing to healthcare expenditures (9). Moreover, Another study conducted in 133 countries showed that, in just 5 years (i.e., from 2005 to 2010), CHE rose from 9.7 to 11.7% (10). Although many studies have examined CHE, most focused on whole populations, not specific vulnerable groups.

In China, owing to the complexity and severity of breast cancer, the costs associated with its diagnosis and treatment are usually higher than those of other diseases, showing a high OOP expenditure and leading to countless CHE incidents. Nationally, the basic health insurance system comprises the Urban Employee Basic Medical Insurance (UEBMI) and the Urban and Rural Resident Basic Medical Insurance (U&RRBMI), with the first offering a higher reimbursement rate compared to the latter (11). However, the reimbursement rate of UEBMI is only 55%.

Despite these facts, few studies have evaluated CHE, its influencing factors in households with breast cancer patients, and the impact of different insurances on CHE in China. Thus, we aimed to provide new information about the extent of the effect and the relevant factors of CHE in households with breast cancer patients; furthermore, we aimed to explore the ability of the medical insurance to protect Chinese residents from CHE. Our findings may contribute to improving and adjusting the related healthcare policies, which may evoke a reduced economic burden of disease on families affected by breast cancer.

## Methods

### Context

The Cancer Screening Program in Urban China (CanSPUC), a key national project supported by the Chinese government, was initiated in 2012. It has served to conduct evaluations of personal cancer risk, clinical cancer screening, health economic evaluation, and follow-up work during the past years. Specifically, it primarily aimed to explore how to appropriately implement the screening of high risk populations and the early diagnosis of major cancers in Chinese urban populations. By 2018, it covered 20 provinces/municipalities in China (12).

### Data Collection

A multicenter cross-sectional survey was conducted from January 2018 to June 2019 as part of CanSPUC. Geographic regions/provinces were grouped into eastern, central and western in line with the classification of economic development zones by the Chinese National Bureau of Statistics. Nine tertiary hospitals, were selected from these zones considering cancer patient volumes and completeness of medical records, including Guangdong Cancer Hospital (eastern); Anhui Cancer Hospital, Heilongjiang Cancer Hospital, Shanxi Cancer Hospital (central); Guangxi Cancer Hospital, Yunnan Cancer Hospital, the Regional Cancer Hospital and two city hospitals in Inner Mongolia (western).

Cancer patients diagnosed initially over the period from 01 January 2015 to 31 December 2016 were eligible for CanSPUC program. Eligible study participants were identified from the hospital records and then approached for a survey. The questionnaire was administered through face-to-face interviews. The survey was coordinated by the National Cancer Center. The interviewers were trained prior to deployment and required to check completeness of the questionnaire before concluding each interview. The questionnaire for this survey collected data regarding demographic characteristics, household income and expenditure, medical expenses for cancer treatment, and insurance compensation. A total of 2,565 patients were investigated, including lung cancer, breast cancer, colorectal cancer, oesophageal cancer, stomach cancer and liver cancer.

Regarding household income and expenditure, respondents were asked to describe them for both 2015 and 2016. Regarding medical expenses for cancer treatment, respondents were asked to describe this variable over a 2-year period (i.e., 2 months before and 22 months after the diagnosis); these costs included payments for hospital diagnosis, treatment, and medicines (prescription and non-prescription drugs) purchased from pharmacy retail stores. Household consumption expenditure includes the amount spent on food, clothing and daily necessities, communication and transportation, Housing, water, electricity and fuel, cultural recreation and entertainment, medical care and other consumption spending.

Among these, Breast cancer patients were selected as subjects of this study. Inclusion criteria were as follows: (1) having been diagnosed for the first time with primary breast cancer, (2) having been initially diagnosed between 1 January 2015 and 31 December 2016, and (3) having subsequently received cancer treatment. The exclusion criterion was having cancers in multiple organs. In total, 639 households that had a breast cancer patient were selected, including Guangdong (19.9% of the total sample), Anhui (7.2%), Heilongjiang (12.1%), Shanxi (14.4%), Guangxi (12.1%), Yunnan province (17.9%), and Inner Mongolia (16.4%). Regarding household income and expenditure, owing to difficulties in articulating a clear cut-off point for the income and expenditure data, we calculated average income and expenditure across the 2 years to match the breast cancer treatment cost data. Regarding medical expenses, we calculated average medical expenses across the 2 years.

Data were double-entered into EpiData 3.1 to ensure accuracy and analysed using Stata 15.

### Measuring CHE

Based on prior studies (5, 6, 13), CHE was defined as OOP equal to or greater than a threshold level of 40% of household's CTP. In this research, OOP covered only direct medical expenditures. CTP was defined as households' non-food expenditure; we also used CTP as the denominator to calculate CHE, which allowed us to partly avoid measurement bias; such bias is often neglected when examining poverty-ridden families. Based on prior research (14, 15), we used the indicator E to demonstrate whether CHE occurred. In the equation below, the indicator *Ei* defined whether CHE occurred:

$$\begin{array}{l} Ei=\{\begin{array}{c} 0,\;\frac{oop}{xi-f(x)}<Z\\ 1,\;\frac{oop}{xi-f(x)}>Z \end{array} \end{array}$$

Where *i* is household *i, xi* is the total consumption expenditure, *f(x)* is the food expenditure, and *Z* is the threshold of CHE. Based on this definition and prior research (14, 15), the incidence and intensity were calculated as follows:

$$\begin{array}{l} Headcount=\frac{1}{N}\sum_{i=1}^{N}Ei\\ Overshoot=\frac{1}{N}\sum_{i=1}^{N}Ei(\frac{oop}{xi-f\left(x\right)}-Z)\\ \;MPO=\frac{Overshoot}{Headcount} \end{array}$$

In these formulas, *N* is the sample size. Headcount was CHE incidence, which is the percentage of households with breast cancer patient whose OOP expenditures exceeded the given threshold. Overshoot is the average distance, it reflects the intensity of CHE in the overall households with cancer patients. MPO is the mean relative distance, it indicates the intensity of CHE in the households suffering from CHE (14, 16).

### Statistical Analysis

The demographic profile of the breast cancer patients was described using the frequency and percentage for categorical variables (e.g., education level, marital status, and insurance type). The chi-squared test was applied to examine the associations between CHE and other variables including gender, age, education level, marital status, course of disease, household size, insurance, income level and treatment method. The statistically significant variables were then entered into the multivariate logistic regression. *P* < 0.05 was considered statistically significant.

A study has shown that the asymptotic quality of the standard maximum likelihood (ML) model can eliminate sample size problems (17); however, some researchers, specifically Hart and Clark (18, 19), suggested that reasoning problems began to occur when the sample size was < 30; Eliaison, in turn, recommended a sample size of higher than 60 participants. Therefore, our sample size, which comprised 639 households with breast cancer patients, was adequate to meet the aforementioned requirements.

## Results

The general characteristics of breast cancer patients and their families are shown in Table 1. Overall, 0.78% patients were male and 99.22% female, and their average age was 53.9 years [importantly, a study showed that the annual incidence of male breast cancer amounts to <1% of all breast cancer patients (20)]. Most patients completed junior and senior high school (53.68% of the total sample), followed by university and higher degrees (25.35%), primary school (16.43%), and none (4.54%). Regarding marital status, 88.11% of the respondents were married. Regarding disease course, 88.26% of the respondents had cancer for 1–2 years. Regarding household size, 59% of the households comprised less than four people. Regarding type of medical security, those under the UEBMI were 49.61% of the sample, and under the U&RRBMI were 47.42%. Regarding treatment method, surgery alone accounted for 34.43% of the sample and surgery and postoperative adjuvant chemotherapy accounted for 35.84%.

**Table 1:** Participant characteristics.

**Sociodemographic characteristics**	**Number**	**Percent (%)**
**Gender**		
Male	5	0.78
Female	634	99.22
**Age (years)**		
≤ 60	467	73.08
>60	172	26.92
**Education level**		
None	29	4.54
primary school	105	16.43
Junior and Senior high school	343	53.68
University and above	162	25.35
**Marital status**		
Married	563	88.11
Rest 1	76	11.89
**Course of disease**		
<1 year	75	11.74
1–2 year	564	88.26
**Household size**		
≤ 3	377	59.00
>4	262	41.00
**Insurance**		
UEBMI	317	49.61
U&RRBMI	303	47.42
Rest 2	19	2.97
**Income level (RMB)**		
≤ 80,000	444	69.48
>80,000	195	30.52
**Treatment method**		
Surgery alone	220	34.43
Radical surgery	101	15.81
Radiotherapy alone or chemotherapy alone	52	8.14
Surgery and postoperative adjuvant chemotherapy	229	35.84
Neoadjuvant chemotherapy and surgery	19	2.97
Rest 3	18	2.82

*Rest 1 represents unmarried, divorced and widowed. Rest 2 stands for Public medical, commercial insurance and uninsured. Rest 3 stands for symptomatic treatment, concurrent radiotherapy and chemotherapy and oral medication. The UEBMI represents Urban Employee Basic Medical Insurance. UEBMI is a government-run mandatory insurance program based on employment. Because there is no guarantee for urban residents without formal employment, the government launched the Urban Resident Basic Medical Insurance (URBMI) plan in 2007. For uninsured rural residents, the New Cooperative Medical Scheme (NCMS) was implemented in 2003. U&RRBMI includes URBMI and NCMS. The RMB is the abbreviation of Ren Min Bi, which is the currency of China*.

**Figure 1:**
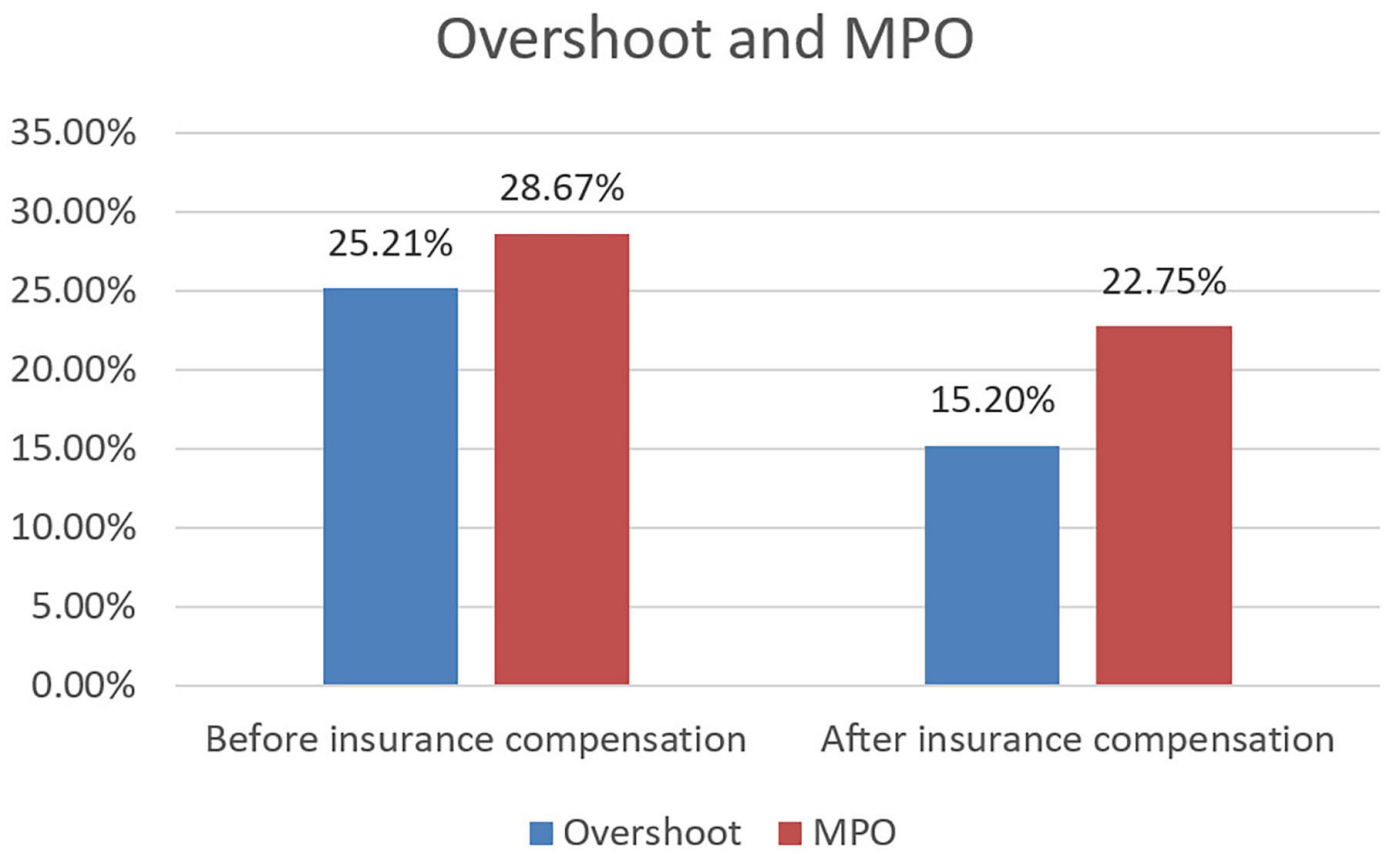
Intensity of CHE before and after insurance compensation. The Overshoot is the average distance, it reflects the intensity of CHE in the overall households with cancer patients. The MPO represents mean relative distance, it indicates the intensity of CHE in the households suffering from CHE.

CHE incidence and intensity—defined by Headcount and Overshoot—for all households before and after insurance compensation are depicted in Table 2 and Figure 1. The Headcount of households with breast cancer patients before insurance compensation was 87.95%; after compensation, it was 66.82% (*p* = 0.000). Thus, insurance was shown to reduce the incidence of CHE by 21.13%.

**Table 2:** Incidence of CHE before and after insurance compensation.

**Sociodemographic characteristics**	**CHE**		
	**Before insurance compensation**	**After insurance compensation**	***p***
**Gender**			
Male	100.00%	60.00%	0.745
Female	87.85%	66.88%	
**Age (years)**			
≤ 60	87.58%	66.81%	0.990
>60	88.95%	66.86%	
**Education level**			
None	89.66%	86.21%	0.000
primary school	91.43%	76.19%	
Junior high school and Senior high school	89.21%	67.64%	
University and above	82.72%	55.56%	
**Marital status**			
Married	87.39%	66.61%	0.753
Rest 1	92.11%	68.42%	
**Course of disease**			
<1 year	81.33%	52.00%	0.004
1–2 year	88.83%	68.79%	
**Household size**			
≤ 3	88.06%	64.46%	0.127
>4	87.79%	70.23%	
**Insurance**			
UEBMI	85.80%	58.04%	0.000
U&RRBMI	90.43%	75.91%	
Rest 2	84.21%	68.42%	
**Income level (RMB)**			
≤ 80,000	91.89%	74.77%	0.000
>80,000	78.97%	48.72%	
**Treatment method**			
Surgery alone	82.27%	57.73%	0.000
Radical surgery	84.16%	56.44%	
Radiotherapy alone or chemotherapy alone	90.38%	73.08%	
Surgery and postoperative adjuvant chemotherapy	93.89%	76.42%	
Neoadjuvant chemotherapy and Surgery	94.74%	84.21%	
Rest 3	88.89%	77.78%	
**Total**	87.95%	66.82%	

Meanwhile, the Headcount of households with breast cancer patients protected by the UEBMI were 85.80 and 58.04% before and after insurance compensation (*p* = 0.000), respectively; Thus, the UEBMI was shown to reduce the incidence of CHE by 27.76%. The Headcount of households with breast cancer patients protected by the U&RRBMI were 90.43 and 75.91% (*p* = 0.000) before and after insurance compensation, respectively; thus, the U&RRBMI was shown to reduce the incidence of CHE by 14.52%.

As shown in Figure 1, the MPO were 28.67 and 22.75% before and after insurance compensation, respectively. Moreover, households that faced CHE at 40% of the total expenditure averagely devoted 62.75% (22.75 + 40%) of their earnings to afford health services. The Overshoot of households with breast cancer patients were 25.21 and 15.20% before and after insurance compensation, respectively. Breast cancer households that actually had experienced catastrophe at the 40% threshold spent an average of 22.75% (MPO) over this threshold. Thus, these households spent an average of 62.75% of all non-food expenditures on OOP medical expenses.

Patients with breast cancer who had no education, longer course of disease, U&RRBMI insurance, lower income level and more expensive treatment method tended to have higher incidence of CHE than the others (Table 2). The logistic regression model further confirmed that education, course of disease, insurance, income level and treatment method were significant predictors of CHE (Table 3).

**Table 3:** Multivariate logistic regression model of determinants of CHE.

**Determinants**	**OR**	**SE**	**p**	**95% CI**	
**Education level (ref: none)**					
primary school	0.299	0.184	0.049	0.089	0.996
Junior and Senior high school	0.261	0.152	0.022	0.083	0.821
University and above	0.274	0.168	0.036	0.082	0.916
**Course of disease(ref:** ** <1 year)**					
1–2 year	2.74	0.776	0.000	1.574	4.778
**Insurance (ref: UEBMI)**					
U&RRBMI	1.677	0.368	0.018	1.091	2.579
Rest 2	1.400	0.755	0.532	0.486	4.030
**Income level (ref:** **≤80,000)**					
>80,000	0.306	0.064	0.000	0.202	0.463
**Treatment method (ref: surgery alone)**					
Radical surgery	0.717	0.190	0.210	0.467	1.206
Radiotherapy or chemotherapy	1.724	0.628	0.135	0.843	3.523
Surgery and adjuvant chemotherapy	2.377	0.532	0.000	1.532	3.686
Neoadjuvant chemotherapy and surgery	4.131	2.831	0.038	1.078	15.83
Rest 3	3.131	1.922	0.063	0.940	10.42
**Cons**	0.578	0.703	0.411		

## Discussion

To the best of our knowledge, this was the first study that examined CHE incidence and analysed and compared CHE incidence and intensity before and after insurance compensation among households with breast cancer patients in China. Our research found that OOP expenditures for breast cancer patients led 66.82% of our sample to incur in CHE. The incidence of CHE in our study were significantly higher than Malaysia (at 47.8%) (21), South Korea (39.8%) (22), and eight Southeast Asian countries (48%) (23). Concomitantly, the CHE rates in our study were slightly lower than those in Iran (67.9%) (24). Additionally, the overall CHE incidence in the general population was only of 13.0%, a value confirmed by another Chinese study (25). Thus, OOP expenditures regarding breast cancer treatments impose a serious economic burden on most households with breast cancer patients in China.

Our results also illustrated that insurance is capable of protecting some households with breast cancer patients from the impact of CHE; however, the overall incidence of CHE remained very high. Specifically, the impact of UEBMI on CHE was greater than that of U&RRBMI; particularly, patients with U&RRBMI were more likely to experience CHE. A prior study, which showed that the UEBMI and U&RRBMI have different deductibles, limit lines, and reimbursement rates, may explain this result (11). Therefore, stakeholders and decision-makers regarding breast cancer in China should not only endeavour to expand the scope and depth of these health insurances but also reduce the differences between the UEBMI and U&RRBMI, as this may help relieve patients' and their families' OOP medical expenditures.

Additionally, in our study, the factors associated of CHE in households with breast cancer patients included education, disease course, health insurance type, treatment method, and income. Particularly, having no education, a longer disease course, an expansive treatment method, and low income were factors that had a significant impact on CHE. Concurring with our results, a study conducted in 10 countries in Southeast Asia showed that education, income, and health insurance type were all determinants of CHE (23). They further found that cancer stage also influenced the occurrence of CHE, in that the likelihood of CHE increased as the cancer stage became higher. We deemed that this variable was like the treatment method variable we utilised; thus, cancer stage and treatment methods may affect the OOP expenditure of households with breast cancer patients, thereby affecting the occurrence of CHE.

However, it is hard for researchers and other stakeholders to change the education, disease course, and income variables of households with breast cancer patients; nonetheless, we can enhance the financial protection provided by insurances and relieve treatment costs for breast cancer to help reduce the incidence of CHE. Currently, the coverage of the basic Chinese health care insurances we studied can be deemed as high; however, they have a somewhat limited impact on OOP health expenditures. Particularly, reimbursement rates are the highest for the UEBMI (at 55%), followed by the URBMI (40%), and the New Rural Cooperative Medical Scheme (30%) (11). For households with breast cancer patients, these reimbursements ratios hinder the ability of these insurances to protect people from incurring in CHE. Therefore, the Chinese government should further optimise and adjust these programs to reduce CHE, specifically for households with critical diseases such as breast cancer that are related to high OOP health expenditures.

According to our findings, the CHE rates for households with breast cancer patients owe not only to the low reimbursement levels mentioned above but also to high medical costs; this finding is in line with the literature (26). In China, these high medical costs may be because of insufficient governmental financial input and the fee-for-service payment mechanism (27–30). The Chinese government had taken full responsibility for financing public hospitals at the end of the 1970s. However, the subsidies of government have been reduced to less than one-third of the income of public hospitals in the past three decades. Accordingly, a study showed that public hospitals need to heavily rely on OOP for cost recovery (29). Meanwhile, the fee-for-service methodology has been shown to encourage doctors to provide excessive medical treatments (29, 30). Moreover, the National Bureau of Statistics showed that the national health expenditure per capita increased from 1,314 Yuan in 2009 to 4,236 Yuan in 2018 (31). Therefore, policy-makers should endeavour to develop policies that help control the cost of medical procedures; we suggest potential reforms in the insurance payment method to reduce patients' economic loss.

Our study has several limitations. First, we defined OOP regarding only direct medical expenditures, excluding direct non-medical costs. Second, we evaluated CHE only in households that received treatment, excluding households that could not afford the treatment; thus, these variables in households with breast cancer patients might, to some extent, have been underestimated. Third, owing to data availability, we did not include not all potential influencing factors of CHE, such as some unobservable household characteristics; this may lead to potentially biassed estimation of the influencing factors of CHE.

## Conclusions

OOP health expenditure for breast cancer patients was shown to be financially catastrophic for most households in our sample. Moreover, medical insurance was shown to protect some Chinese households with breast cancer patients from the impact of CHE. However, the insurance reimbursement rates were relatively low, and the overall CHE incidence remained very high. Some socioeconomic and non-socioeconomic factors were shown to significantly affect CHE in the studied households. Albeit we cannot change these non-socioeconomic factors, policies aimed at reducing CHE can address the socioeconomic factors related to healthcare outcomes in households of patients with critical diseases such as breast cancer.

## Data Availability Statement

The data of the current study are available from the Chinese Academy of Medical Sciences upon reasonable request. Requests to access these datasets should be directed to lgx6301@163.com.

## Ethics Statement

This study was approved by the Ethics Committee of the Cancer Institute and Hospital, Chinese Academy of Medical Sciences (15-070/997).

## Author Contributions

JH and W-qC: acquisition of data and conceived the research idea. J-fS, W-qF, and XZ: conception and design, analysis and interpretation of data. C-yS: writing and drafting of the manuscript, analysis and interpretation of data, and statistical analysis. G-xL: critical revision of the manuscript for important intellectual content, and conceived the research idea. All authors have read and approved the final manuscript.

## Conflict of Interest

The authors declare that the research was conducted in the absence of any commercial or financial relationships that could be construed as a potential conflict of interest.

## References

[1] Bray F Ferlay J Soerjomataram I Siegel RL Torre LA Jemal A. Global cancer statistics 2018: GLOBOCAN estimates of incidence and mortality worldwide for 36 cancers in 185 countries. CA Cancer J. Clin. (2018) 68:394–424. 10.3322/caac.21492 30207593

[2] Cancer incidence and mortality in China 2014. Chin J Cancer Res. (2018) 30:1–12. 10.21147/j.issn.1000-9604.2018.01.01 PMC5842223 29545714

[3] Reinhardt U Cheng T. The world health report 2000 – health systems: improving performance. Bull World Health Org. (2000) 78:1064. Available online at: https://www.science-open.com/document?vid=0c51da9c-de25-40a8-82ee-9f558167f03311910962

[4] Xu K Evans DB Kawabata K Zeramdini R Klavus J Murray CJL. Distribution of Health Payments and Catastrophic Expenditures. Methodology. Discussion paper 2. WHO (2005). Available online at: https://apps.who.int/iris/discover?query=Distribution+of+health+payments+and+catastrophic+expenditures+Methodology

[5] Xu Y Gao J Zhou Z Xue Q Yang J Luo H. Measurement and explanation of socioeconomic inequality in catastrophic health care expenditure: evidence from the rural areas of Shaanxi Province. BMC Health Serv Res. (2015) 15:256. 10.1186/s12913-015-0892-2 26138738 PMC4490607

[6] Si Y Zhou Z Su M Ma M Xu Y Heitner J. Catastrophic healthcare expenditure and its inequality for households with hypertension: evidence from the rural areas of Shaanxi Province in China. Int J Equity Health. (2017) 16:27. 10.1186/s12939-016-0506-6 28666448 PMC5493855

[7] Van Minh H Kim Phuong NT Saksena P James CD Xu K. Financial burden of household out-of pocket health expenditure in Viet Nam: findings from the national living standard survey 2002-2010. Soc Sci Med. (2013) 96:258–63. 10.1016/j.socscimed.2012.11.028 23246399

[8] Zhou C Long Q Chen J Xiang L Li Q Tang S. The effect of NCMS on catastrophic health expenditure and impoverishment from tuberculosis care in China. Int J Equity Health. (2016) 15:172. 10.1186/s12939-016-0463-0 27756368 PMC5069881

[9] Xu K Evans DB Carrin G Aguilar-Rivera AM Musgrove P Evans T. Protecting households from catastrophic health spending. Health Affairs. (2007) 26:972–83. 10.1377/hlthaff.26.4.972 17630440

[10] Rao KD Makimoto S Peters M Leung GM Bloom G Katsuma Y. Vulnerable populations universal health coverage. In: Kharas H, McArthur JW, Ohno I, editors. Leave No One Behind. Brookings Institution Press (2020). p. 129–48.

[11] Li Y Wu Q Xu L Legge D Hao Y Gao L. Factors affecting catastrophic health expenditure and impoverishment from medical expenses in China: policy implications of universal health insurance. Bull World Health Org. (2012) 90:664. 10.2471/BLT.12.102178 22984311 PMC3442391

[12] Chen WQ Li N Shi JF Ren JS Chen HD Li J. Progress of Cancer Screening Program in Urban China. Chinese Academy of Medical Sciences; China Cancer (2019).

[13] Xu K Evans DB Kawabata K Zeramdini R Klavus J Murray CJ. Household catastrophic health expenditure: a multicountry analysis. Lancet. (2003) 362:111–7. 10.1016/S0140-6736(03)13861-5 12867110

[14] Wagstaff A van Doorslaer E. Catastrophe and impoverishment in paying for health care: with applications to Vietnam 1993-1998. Health Econ. (2010) 12:921–33. 10.1002/hec.776 14601155

[15] Wang Z Li X Chen M. Catastrophic health expenditures and its inequality in elderly households with chronic disease patients in China. Int J Equity Health. (2015) 14:8. 10.1186/s12939-015-0134-6 25599715 PMC4304672

[16] Ghiasvand H Abolghasem Gorji H Maleki M Hadian M. Catastrophic health expenditure among iranian rural and urban households, 2013 - 2014. Iran Red Crescent Med J. (2015) 17:e30974. 10.5812/ircmj.30974 26473081 PMC4601211

[17] Karaca-Mandic P Norton EC Dowd B. Interaction terms in non-linear models. Health Serv Res. (2011) 47:255–74. 10.1111/j.1475-6773.2011.01314.x 22091735 PMC3447245

[18] Eliason SR. Maximum Likelihood Estimation: Logic and Practice. Sage Publications (1993).

[19] Hart RA Clark DH. Does size matter? Exploring the small sample properties of maximum likelihood estimation. In*: Annual Meeting of the Midwest Political Science Association* (1999).

[20] Fentiman Ian S Fourquet Alain Hortobagyi Gabriel N. Male breast cancer. Lancet. (2006) 367:595–604. 10.1016/S0140-6736(06)68226-3 16488803

[21] Azzani M Yahya A Roslani AC Su TT. Catastrophic health expenditure among colorectal cancer patients and families: a case of Malaysia. Asia Pac J Public Health. (2017) 29:485–94. 10.1177/1010539517732224 29019257

[22] Choi JW Cho KH Choi Y Han KT Kwon JA Park EC. Changes in economic status of households associated with catastrophic health expenditures for cancer in South Korea. Asian Pacific J Cancer Prevent. (2014) 15:2713–7. 10.7314/APJCP.2014.15.6.2713 24761890

[23] ACTION Study Group Kimman M Jan S Yip CH Thabrany H Peters SA. Catastrophic health expenditure and 12-month mortality associated with cancer in Southeast Asia: results from a longitudinal study in eight countries. BMC Med. (2015) 13:1–11. 10.1186/s12916-015-0433-1 26282128 PMC4539728

[24] Kavosi Z Rashidian A Pourreza A Majdzadeh R Pourmalek F Hosseinpour AR. Inequality in household catastrophic health care expenditure in a low-income society of Iran. Health Policy Plan. (2012) 27:613–23. 10.1093/heapol/czs001 22279081

[25] Wu QH Li Y Xu L Hao YH. Effect of health insurance on reduction of catastrophic health expenditure in China. Chin J Health Policy. (2012) 05:626. 10.3969/j.issn.1674-2982.2012.09.012

[26] Zhang L Cheng X Tolhurst R Tang S Liu X. How effectively can the new cooperative medical scheme reduce catastrophic health expenditure for the poor and non-poor in rural China? Trop Med Int Health. (2010) 15:468–75. 10.1111/j.1365-3156.2010.02469.x 20180938

[27] Blumenthal D Hsiao W. Privatization and its discontents–the evolving Chinese health care system. New Engl J Med. (2005) 353:1165–70. 10.1056/NEJMhpr051133 16162889

[28] Hu S Tang S Liu Y Zhao Y Escobar ML de Ferranti D. Reform of how health care is paid for in China: challenges and opportunities. Lancet. (2008) 372:1846–53. 10.1016/S0140-6736(08)61368-9 18930520

[29] Ramesh M. Reasserting the role of the state in the healthcare sector: lessons from Asia. Policy Soc. (2009) 27:129–36. 10.1016/j.polsoc.2008.09.004

[30] Yip W Hsiao WC. The Chinese health system at a crossroads. Health Affairs. (2008) 27:460–8. 10.1377/hlthaff.27.2.460 18332503

[31] Colditz GA Atwood KA Emmons K Monson RR Willett WC Trichopoulos D. Harvard report on cancer prevention volume 4: Harvard cancer risk index. Risk index working group, harvard center for cancer prevention. Cancer Causes Control. (2000) 11:477–88. 10.1023/A:1008984432272 10880030

